# Epigenetic Regulation of Excitatory Amino Acid Transporter 2 in Neurological Disorders

**DOI:** 10.3389/fphar.2019.01510

**Published:** 2019-12-13

**Authors:** Mohammad Afaque Alam, Prasun K. Datta

**Affiliations:** Department of Neuroscience, Center for Comprehensive NeuroAIDS, Lewis Katz School of Medicine at Temple University, Philadelphia, PA, United States

**Keywords:** glutamate, microRNA, excitatory amino acid transporter 2, DNA methyltransferase, histone deacetylase, CRISPR/Cas9

## Abstract

Excitatory amino acid transporter 2 (EAAT2) is the predominant astrocyte glutamate transporter involved in the reuptake of the majority of the synaptic glutamate in the mammalian central nervous system (CNS). Gene expression can be altered without changing DNA sequences through epigenetic mechanisms. Mechanisms of epigenetic regulation, include DNA methylation, post-translational modifications of histones, chromatin remodeling, and small non-coding RNAs. This review is focused on neurological disorders, such as glioblastoma multiforme (GBM), Alzheimer’s disease (AD), amyotrophic lateral sclerosis (ALS), Parkinson’s disease (PD), bipolar disorder (BD), and neuroHIV where there is evidence that epigenetics plays a role in the reduction of EAAT2 expression. The emerging field of pharmaco-epigenetics provides a novel avenue for epigenetics-based drug therapy. This review highlights findings on the role of epigenetics in the regulation of EAAT2 in different neurological disorders and discusses the current pharmacological approaches used and the potential use of novel therapeutic approaches to induce EAAT2 expression in neurological disorders using CRISPR/Cas9 technology.

## Introduction

**Figure 1:**
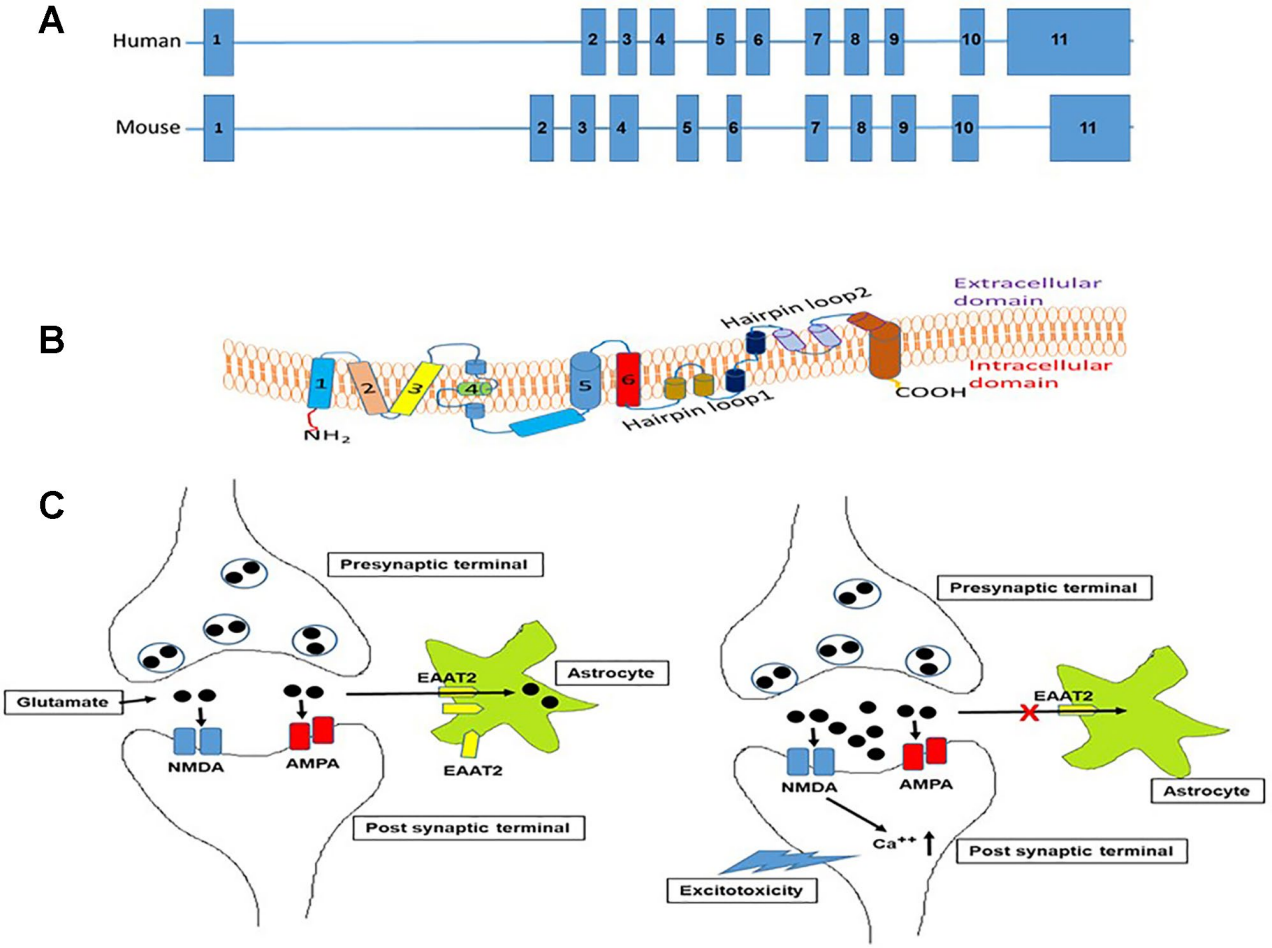
**(A)**. Schematic representation of the organization of introns and exons of SLC1A2 gene in human and mouse based on UCSC genome browser. The size of SLC1A2 gene is 11704 bp in human and 11565 bp in mouse. **(B)**. Schematic depiction of the organization of EAAT2 in the plasma membrane as deduced from crystallographic data (Yernool et al., 2004) and adapted from Boston-Howes et al. (2006). The protein contains eight transmembrane domains and two helical hairpin loops (HP1) and (HP2). These hairpin structures are involved in transport of amino acids mainly -glutamate. **(C)**. Schematic representation of the mechanism of glutamate-mediated excitotoxicity in the synaptic cleft due to dysregulation of EAAT2 expression in astrocytes. In normal scenario, depolarization of nerve terminal (presynaptic) glutamate is released from synaptic vesicles. Released glutamate then binds to ionotropic glutamate receptors (NMDA-R and AMPA-R) on the postsynaptic terminal that results in depolarization and action potential generation. Glutamate is then removed quickly from the synaptic cleft by astrocyte EAAT2 transporter to prevent the overstimulation of glutamate receptors. However, excessive glutamate accumulation in the synaptic cleft due to dysregulation of astrocyte EAAT2 expression causes overstimulation of NMDA and AMPA receptors that results in the build-up of intracellular Ca^++^ ions leading to neuronal death or excitotoxicity.

The human excitatory amino acid transporter 2 (EAAT2) or glutamate transporter 1 (GLT-1) in the rodents is the primary glutamate transporter in the astrocytes (Rothstein et al., 1996; Sheldon and Robinson, 2007; Kim et al., 2011), and handles 90% of total glutamate uptake in the CNS (Tanaka et al., 1997). The SLC1A2 (solute carrier family, member 2) gene, located on chromosome codes for EAAT2 in humans (Meyer et al., 1997), while in mouse, it is located on chromosome 2 and is known as glutamate transporter 1 (GLT1). The size of the human and mouse EAAT2 gene is ∼11.7 and ∼11.5 kb, respectively, and both genes contain 11 exons (**Figure 1A**).

The promoter analysis of the human EAAT2 gene reveals that it harbors transcription factor binding sites for Nuclear factor kappa-B (NFκB), Specificity protein 1 (Sp1), cAMP responsive element binding protein (CREB), Ying-yang 1 (YY1), and peroxisome proliferator activated receptor (PPAR) response element (Su et al., 2003; Sitcheran et al., 2005; Zschocke et al., 2007; Romera et al., 2007; Allritz et al., 2010; Unger et al., 2012; Karki et al., 2014; Vartak-Sharma et al., 2014). Also, the proximal promoter harbors CpG islands at position −1472 to −1146 and −680 to −494, with 17 CpG and 15 CpG dinucleotides, respectively (Zschocke et al., 2007). The human EAAT2 cDNA harbors an unusually long 3’-UTR of 9684 bp (Kim et al., 2003). Sequence analysis shows that the 3'-UTR of EAAT2 cDNA is nearly identical and conserved in human, macaque, rat, and mouse (Kim et al., 2003). This observation suggests that it is likely that EAAT2 mRNA expression can be regulated at the post-transcriptional level by miRNAs.

EAAT2 is a plasma membrane sodium-dependent, high-affinity amino acid transporter that mediates the uptake of L-glutamate (Arriza et al., 1994). In brief, the protein has eight transmembrane domains with the amino- and carboxy-terminal located intracellularly (**Figure 1B**). It clears the excitatory neurotransmitter glutamate from the extracellular space at synapses in the brain (Rothstein et al., 1996). Glutamate clearance by astrocyte is critical for proper synaptic activation, and also glutamate is converted to glutamine and transported out of the astrocytes into neurons for reuse in glutamate synthesis (Erecińska and Silver, 1990). Furthermore, reuptake of glutamate by EAAT2 also prevents neuronal damage caused by excessive activation of NMDA receptors (**Figure 1C**), a phenomenon known as excitotoxicity (Olney and Sharpe, 1969).

## Epigenetic Regulators: The Writers, Readers, and Erasers

Epigenetics is defined as changes in gene expression without the involvement of changes in the DNA sequence. The epigenetic “writers” are enzymes such as DNA methyltransferases, histone lysine methyltransferases, protein arginine methyltransferases, and histone acetyltransferases that catalyze the addition of a functional group to a protein or nucleic acid (Gillette and Hill, 2015). The epigenetic “readers” are proteins or enzymes such as methyl CpG binding proteins, histone methylation readers, and histone acetylation readers that recognize methylated DNA, methylated lysine residues in proteins and acetylated histones, respectively. The epigenetic “erasers” are enzymes, such as ten-eleven translocation (TET) family of proteins, histone demethylases, and histone deacetylases (HDAC) that demethylate DNA, demethylate lysine residues on histone proteins, and deacetylate histone proteins (see reviews; Gillette and Hill, 2015; Biswas and Rao, 2018).

## DNA Methyltransferases (DNMTs)

DNMTs are classified into three categories, DNMT1, DNMT2, DNMT3 [DNMT3a, DNMT3b, and DNMT3L] (Lyko, 2018; Gujar et al., 2019). DNMT1 is involved in the maintenance methylation (Ren et al., 2018). DNMT3a and DNMT3b methylate cytosine residues in CpG island(s) and considered as *de novo* methyltransferases. DNMT1, DNMT3a, and DNMT3b catalyze the addition of a methyl group from S-adenosylmethionine (SAM) to cytosine resulting in 5-mC. 5-mC acts as a stable transcriptional repressor (Kitsera et al., 2017). DNMT2 and DNMT3L are non-canonical family members, as they do not possess catalytic DNMT activity (Lyko, 2018).

## Ten-Eleven Translocation

DNA demethylation involves the TET family of methylcytosine dioxygenases that are α-KG-dependent enzymes (Koivunen and Laukka, 2018). This family consists of TET1, TET2, and TET3, which participate in the conversion of 5-mC to 5-hmC to promote reversal of methylation (Ito et al., 2010; Melamed et al., 2018). Besides, studies have shown that Tet enzymes also catalyze the conversion of 5-hmC to 5-formylcytosine (5-fC), and 5-carboxylcytosine (5-caC). These modifications serve as DNA demethylation intermediates and are subject to deamination, glycosylase-dependent excision, and repair, resulting in a reversion to unmodified cytosine (Antunes et al., 2019).

## DNMT Expression in Astrocytes

In late-stage embryonic development in the brain, DNMT3a is ubiquitously expressed, while DNMT3b expression level decreases but remains high in comparison to early-stage embryos (Okano et al., 1999). The expression of DNMT1 and DNMT3a has been documented in rat brain cortical astrocytes (Zhang et al., 2014).

## TET Expression in Astrocytes

In the brain, NeuN positive neuronal cells express all forms of TETs (Kaas et al., 2013; Li et al., 2014). These observations are tune with reports that neuronal cells are enriched for 5hmC (Szulwach et al., 2011). TET1 expression has been documented in glial fibrillary acidic protein (GFAP) positive astrocytes in the adult mouse hippocampus (Kaas et al., 2013). It has been observed that TET enzymatic activity is inhibited by increased production of 2-hydroxyglutarate in gliomas as a consequence of oncogenic mutations in the metabolic regulators IDH1 (isocitrate dehydrogenase 1) and IDH2 (isocitrate dehydrogenase 2) (Reiter-Brennan et al., 2018).

## Histone Deacetylases

HDACs based on their amino acid sequence, organization of the domains, and catalytic dependence are grouped into four classes (de Ruijter et al., 2003). Class I, II, and IV HDACs are zinc-dependent, while class III are nicotinamide adenine dinucleotide (NAD+) dependent. The class I HDACs include HDAC1, -2, -3, and -8, while class II includes HDAC4, -5, -6, -7, -9, and -10, and class IV is represented by HDAC11 (de Ruijter et al., 2003). Class III HDACs include sirtuins 1–7 (SIRT1–7) that are structurally unrelated to the other HDACs (Carafa et al., 2016).

## HDAC Expression in Astrocytes

A comprehensive study was the first to demonstrate the expression of HDACs in rat brain using high-resolution *in situ* hybridization (ISH) coupled with immunohistochemistry in astrocytes, oligodendrocytes, neurons, and endothelial cells (Broide et al., 2007). The study showed that GFAP-positive astrocytes expressed HDAC3 to HDAC11 (Broide et al., 2007). However, a recent study reported that only HDAC1, 2, and 4 are expressed in rat astrocytes (Kalinin et al., 2013). HDAC 1, 2, 3, and 8 are expressed in normal human astrocytes, and glioblastoma multiforme (GBM) derived astrocytic cell lines (Zhang et al., 2016).

## Sirtuins Expression in Astrocytes

Among the class III HDACs, SIRT1 is the most conserved member of the sirtuin family of NAD+ dependent protein deacetylases (Cohen et al., 2004) and is predominantly a nuclear enzyme but also present in the mitochondria (Tang, 2016). SIRT1 is expressed in mouse (Li M et al., 2018), rat, and human astrocytes (Hu et al., 2017). SIRT2 is a cytoplasmic enzyme (Braidy et al., 2015), and its expression was observed in rat hippocampus and cerebral cortex. Unlike SIRT1, which is primarily a nuclear enzyme SIRT3, 4, 5 are mitochondrial enzymes (Jęśko et al., 2017; Sidorova-Darmos et al., 2018). The expression of SIRT3 was shown in rat astrocytes (Li X et al., 2018). SIRT4 is highly expressed in rat astrocytes (Komlos et al., 2013). It is reported that SIRT5 is expressed in rat striatum (Omonijo et al., 2014). Not much is known about the astrocyte-specific expression of SIRT6 and SIRT7, that are nuclear enzymes except for that fact that they are expressed in rat hippocampus and cerebral cortex (Braidy et al., 2015).

## Noncoding RNA: miRNAs

miRNAs are small noncoding RNAs (20–22 nucleotides) regulate gene expression by binding to seed sequences located in the 3'-UTR of mRNAs (He and Hannon, 2004; Bartel, 2009). The complementarity between the miRNA seed sequence and its target mRNA determines the fate of the mRNA resulting in either translational repression or mRNA cleavage (Guo et al., 2010). A single miRNA can regulate many different mRNAs or can bind to a single site or multiple sites within the 3'-UTR of the mRNA.

## Neurological Disorders and EAAT2 Expression

**Figure 2:**
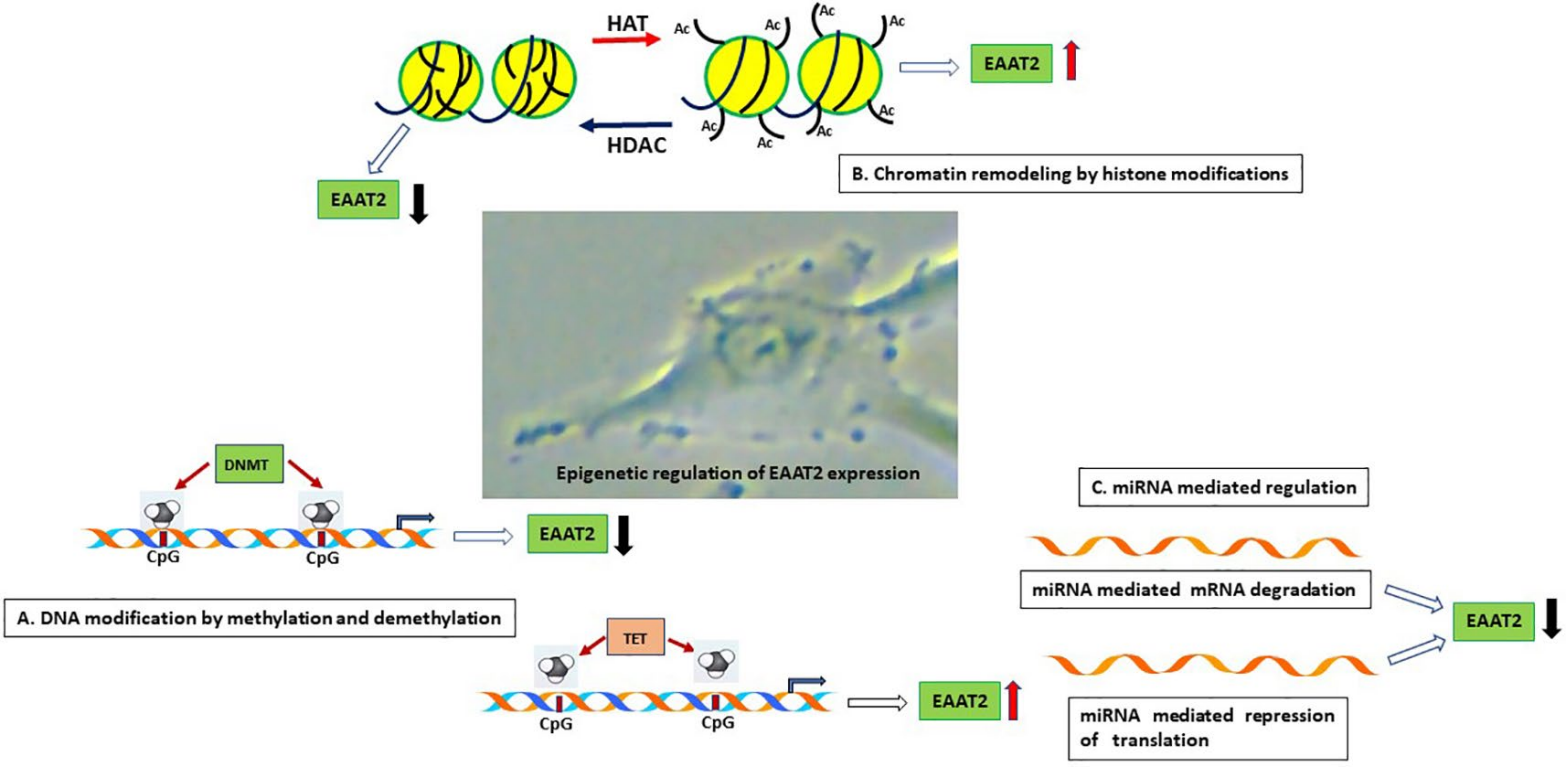
Schematic representation of the epigenetic mechanisms that are involved in astrocyte EAAT2 expression. **(A)**. DNA modification by methylation and demethylation. Hypermethylation of CpG islands on the EAAT2 promoter can repress transcription of EAAT2 gene by inhibiting binding of transcription factors. On the contrary, demethylation of DNA in CpG island can promote transcription factor interaction with DNA and EAAT2 gene transcription. **(B)**. Chromatin remodeling by histone modifications. The observation that HDAC inhibitors induce EAAT2 expression demonstrate that HDAC activity inhibits EAAT2 expression, on the other hand, histone acetylation by HATs can lead to open chromatin and increased accessibility of transcription factors to open chromatin and induce EAAT2 expression. **(C)**. miRNA mediated regulation. Binding of miRNA to the 3’-UTR of EAAT2 mRNA can result in miRNA mediated mRNA degradation or repression of translation resulting in reduced expression of EAAT2. HAT, Histone acetyltransferase; HDAC, Histone deacetylase; DNMT, DNA methyltransferase; TET, Ten eleven translocation enzyme.

In this section, we describe the various neurological disorders where dysregulation of EAAT2 expression have been reported. A summary of the epigenetic changes affecting EAAT2 gene is presented in **Table 1**. The epigenetic changes that are involved in EAAT2 expression is shown in **Figure 2**.

**Table 1:** Epigenetic modifications involved in dysregulation of EAAT2 expression.

Type of Epigenetic modification	Tissue/cell type	Effect on EAAT2/GLT-1	References
**DNA methylation**			
Promoter CpG island methylation	Glioma cell lines.	Reduced EAAT2 mRNA expression.	Zschocke et al., 2007.
Enhanced DNMT1 activity.	Brain tissues of HIV-infected methamphetamine users.	Increase in global DNA methylation.	Desplats et al., 2014.
Hypermethylation of CpG island in promoter.		Not determined.	Desplats et al., 2014.
Hypermethylation of CpG island in promoter region.	Blood DNA.	Not determined	Jia et al., 2017.
CpG site demethylation.	Rat brain astrocytes.	Increase in GLT-1 mRNA expression.	Perisic et al., 2010.
**Histone modifications**			
Overexpression of HDAC1 and -3 (class I), and HDAC6 and -7 (class II).	Rat brain astrocytes.	Decrease in EAAT2 promoter activity.	Karki et al., 2014
Coexpression of HDACs with YY1 or NFκB.	Rat brain astrocytes.	Decrease in EAAT2 promoter activity.	Karki et al., 2014.
HDAC inhibition (SAHA, TSA, Romidepsin).	Rat brain astrocytes.	Increase in EAAT2 promoter activity.	Karki et al., 2014.
HDAC inhibition (TSA).	Glioma cell lines.	Increase in EAAT2 mRNA expression.	Zschocke et al., 2007.
HDAC inhibition (MC1568).	Mouse glia.	Increase in EAAT2 mRNA and protein expression.	Lapucci et al., 2017.
	Spinal cord of rodent model of ALS.	Increase in EAAT2 mRNA and protein expression.	
**Sirtuins**			
SIRT5 knock-out.	Mice brain cortex.	Reduced expression of EAAT2 mRNA.	Koronowski et al., 2018.
**Non coding RNA**			
miR-107.	Nerve cell hypoxia/reoxygenation (H/R) injury.	Inhibition of GLT-1 expression.	Yang et al., 2014.
miR-124a.	Mice neurons.	Induction of GLT-1 expression in astrocytes.	Morel et al., 2013.
miR-124.	Human neural precursor cells and astrocytes.	Induction of EAAT2 expression.	Lee et al., 2014.
miR-218.	Dying motor neurons from rat model of ALS.	Inhibition of EAAT2 expression in astrocytes.	Hoye et al., 2018.
miR-146a	Glioma cell line and human fetal brain astrocytes.	Inhibition of EAAT2 expression in astrocytes.	Deshmane et al., 2018.
**Sumoylation**	Spinal cord astrocytes from SOD1-G93A transgenic mice model of ALS.	Reduced plasma membrane EAAT2 expression due to retention of EAAT2 in the cytoplasm.	Foran et al., 2014.

## Glioblastoma Multiforme (GBM)

GBM, a WHO grade IV astrocytoma is an extremely aggressive, invasive, and destructive primary brain tumor in the adult population (Geraldo et al., 2019). Lee and co-workers demonstrated a strong negative correlation between the expression of Astrocyte Elevated Gene-1 (AEG-1), an oncogene, and EAAT2 by immunofluorescence analyses in human glioma tissue arrays (Lee et al., 2011). Dysregulation of EAAT2 expression is also seen in cell lines derived from tumors (Zschocke et al., 2007; Lee et al., 2011). In two different glioma cell lines, A172 and LN18 that lack EAAT2 expression profiling of DNA methylation by bisulfite sequencing revealed hypermethylation in both CpG islands of EAAT2 promoter (Zschocke et al., 2007).

## Alzheimer’s Disease (AD)

AD is a chronic neurodegenerative disorder that contributes to 60% to 70% of dementia worldwide. Most forms of AD are sporadic, and less than 1% of all cases are familial AD. Early-onset AD is caused by mutations of the genes for APP (amyloid precursor protein), PSEN1 (presenilin 1), and PSEN2 (presenilin 2) (Giau et al., 2019). The pathological hallmarks are β-amyloid plaques localized extracellularly and neurofibrillary tangles, which are localized intracellularly, especially in the frontal cortex and hippocampus (Pinheiro and Faustino, 2019). Studies have shown decreased EAAT2 protein expression in AD brains (Li et al., 1997; Jacob et al., 2007). EAAT2 expression is reduced in astrocytes by oligomeric Aβ by NFAT signaling (Abdul et al., 2009). Dysregulation of EAAT2 expression has been linked in the pathogenesis of AD in APPSw/Ind mice, a transgenic mouse of AD (Takahashi et al., 2015).

## Amyotrophic Lateral Sclerosis (ALS)

ALS is a late-onset and devastating neurodegenerative disorder that is characterized by progressive degeneration of motor neurons in the motor cortex, spinal cord, and brainstem (Verber et al., 2019). Studies have shown that there is a loss of EAAT2 protein in the motor cortex and spinal cord in ALS patients (Rothstein et al., 1995). In the transgenic mice or rats expressing familial ALS-linked mutant SOD1 reduced expression of EAAT2 protein has also been observed (Bruijn et al., 1997; Bendotti et al., 2001; Howland et al., 2002). In addition, another epigenetic modulator, known as sumoylation was shown to regulate localization of EAAT2 expression in SOD1-G93A mouse model of inherited ALS, wherein the cytosolic carboxy-terminal domain is cleaved from EAAT2, conjugated to SUMO1, and results in the accumulation of EAAT2 in the cytoplasm instead of expression in the plasma membrane (Foran et al., 2014).

## Parkinson’s Disease (PD)

PD is a complex neurodegenerative disorder that impacts the dopaminergic neurons located in the midbrain nucleus substantia nigra (Dauer and Przedborski, 2003). The pathological hallmark of PD is the accumulation of α-synuclein oligomers to form Lewy bodies (Wong and Krainc, 2017). In PD, induced in mouse models by 6-hydroxydopamine injection into the nigrostriatal pathway (Chung et al., 2008) and 1-methyl-4-phenyl-1,2,3,6-tetrahydropyridine (Holmer et al., 2005) EAAT2 expression is reduced. Studies have shown that high manganese (Mn) levels induce manganism, symptoms of which are similar to those of PD (Bowman et al., 2011). In this regard, Mn treatment of astrocytes inhibited EAAT2 expression by upregulating YY1 expression that repressed EAAT2 expression at the mRNA and protein level (Karki et al., 2014).

## Bipolar Disorder (BD)

BD is a complex neurobiological disease (Harrison et al., 2018). In BD, both glial cells and neurons are affected and dysregulation of monoamines, altered glutamatergic neurotransmission, increase in oxidative stress, mitochondrial dysfunction, and neuroinflammation play a role in the etiology of the disease (Yuksel and Ongur, 2010; Data-Franco et al., 2017). It is reported that a T-to-G polymorphism in the SLC1A2 gene promoter affects EAAT2 expression in BD (Dallaspezia et al., 2012). A recent study using high resolution melting PCR (HRM-PCR) and thymine-adenine (TA) cloning reported that the SLC1A2 promoter region was hypermethylated in BD (Jia et al., 2017).

## HIV-Associated Neurocognitive Disorder (HAND)

HAND or NeuroHIV persists despite effective antiretroviral therapy (Saylor et al., 2016). HIV-1 and gp120 have been shown to inhibit EAAT2 expression in human fetal brain astrocyte (Wang et al., 2003). Studies using immunohistochemistry have demonstrated that in HAND-positive brain tissues, expression of EAAT2 is reduced in comparison to uninfected brain tissue (Xing et al., 2009). Furthermore, it was shown that treatment of human brain astrocytes with a pro-inflammatory cytokine IL-1β, induced AEG-1 expression that, in turn, upregulated YY1 expression and inhibited EAAT2 transcription (Vartak-Sharma et al., 2014). Elucidation of global DNA methylation status in brain tissues of HIV-individuals who used methamphetamine showed increased levels of DNMT1 activity and also hypermethylation of CpG nucleotides in SLC1A2 promoter (Desplats et al., 2014).

## Role of HDACS and Sirtuins in EAAT2 Expression

Overexpression of HDAC1 and -3 (class I), and HDAC6 and -7 (class II) was shown to inhibit EAAT2 promoter activity in rat astrocytes (Karki et al., 2014). In the same study, the coexpression of HDACs with YY1 or NFκB further attenuated EAAT2 promoter activity (Karki et al., 2014). There is a lack of information on the effect of SIRTs in the regulation of EAAT2 expression. A recent metabolomics study using SIRT5 knock-out mice model showed dysregulation of glutamate levels in brain cortex and reduced expression of EAAT2 at mRNA level (Koronowski et al., 2018).

## Role of miRNA in EAAT2 Expression

The upregulation of miR-107 was shown to inhibit GLT-1 expression in a rodent model of nerve cell hypoxia/reoxygenation (H/R) injury (Yang et al., 2014). Our preliminary studies show that miR-146a reduces EAAT2 expression in U251 cells and human fetal brain astrocytes (Deshmane et al., 2018). A recent report demonstrated that murine neuronal miR-124a induces astroglial EAAT2 not by targeting EAAT2 3’-UTR but by indirectly modulating astrocyte-derived factors that regulate EAAT2 expression (Morel et al., 2013). Also, exosome-mediated delivery of miR-124 was shown to induce the expression of EAAT2 in human neural precursor cells and astrocytes (Lee et al., 2014). In a recent study, a novel mechanism of neurodegeneration in a rat model of ALS was described extracellular miR-218 released from dying motor neurons inhibited EAAT2 expression in astrocytes (Hoye et al., 2018).

## Pharmaco-Epigenetic Strategies to Activate EAAT2 Expression

A successful approach in the treatment of neurodegenerative diseases where epigenetics regulate gene expression could be the use of therapeutic drugs that target epigenetic mechanisms, such as DNA methylation, chromatin, and histone modifications. In this regard, significant advancements have been made to develop drugs that can restore or alter epigenetic mechanisms. In this section, we highlight the findings reported so far with DNMT inhibitors and HDAC inhibitors in the restoration of EAAT2 expression *in vitro* and *in vivo*.

## DNMT Inhibitors

DNMT inhibitors prevent DNA methylation as a consequence reduce promoter hypermethylation, which leads to re-expression of silenced genes. DNMT inhibitors have been widely used as anticancer drugs since hypermethylation of promoters of tumor suppressor genes occurs in numerous cancers (Pfister and Ashworth, 2017). DNMT inhibitors that are approved by the US Food and Drug Administration (FDA) and widely used as anticancer drugs are nucleoside analogs. These are azacytidine (5-aza-deoxycytidine) (Sorm and Vesely, 1964; Christman, 2002) and decitabine (5-aza-2'-deoxycytidine) (Pliml and Sorm, 1964). In this regard, DNMT inhibitor azacytidine was shown to restore EAAT2 expression in a glioma cell line (Zschocke et al., 2007).

## HDAC Inhibitors

Among the four major structural families of HDAC inhibitors viz., short-chain aliphatic acids, hydroxamic acids, benzamides, and cyclic tetrapeptides and depsipeptide only the efficacy of short-chain aliphatic acids, hydroxamic acids, and cyclic tetrapeptides and depsipeptide have been evaluated in inducing EAAT2 expression.

Valproic acid (VPA short-chain aliphatic acid), an FDA-approved anti-epileptic agent and sodium butyrate that inhibits class I and II HDACs (Göttlicher et al., 2001) were reported to prevent manganese-induced inhibition of GLT1 expression in mice (Johnson et al., 2018a; Johnson et al., 2018b). VPA induced CpG site demethylation and acetylated histone H4 enrichment in the distal part of the GLT-1 promoter in rat astrocytes (Perisic et al., 2010).

Hydroxamic acids, Trichostatin A (TSA) (Yoshida et al., 1990) was shown to induce EAAT2 mRNA expression in glioma cells (Zschocke et al., 2007), and EAAT2 promoter activity in rodent astrocyte (Karki et al., 2014).

Suberoylanilide hydroxamic acid (SAHA), an FDA approved drug, also induced EAAT2 promoter activity in rodent astrocyte (Karki et al., 2014).

Among the cyclic tetrapeptides and depsipeptide, Romidepsin has been shown to induce EAAT2 promoter activity (Karki et al., 2014). MC1568, a class II HDAC inhibitor, was reported to upregulate the expression of EAAT2 *in vitro* and also in the spinal cord of SOD1-G93A mice, a rodent model of ALS (Lapucci et al., 2017).

## Potential Use of CRISPR/Cas9 for EAAT2 Gene Expression

**Figure 3:**
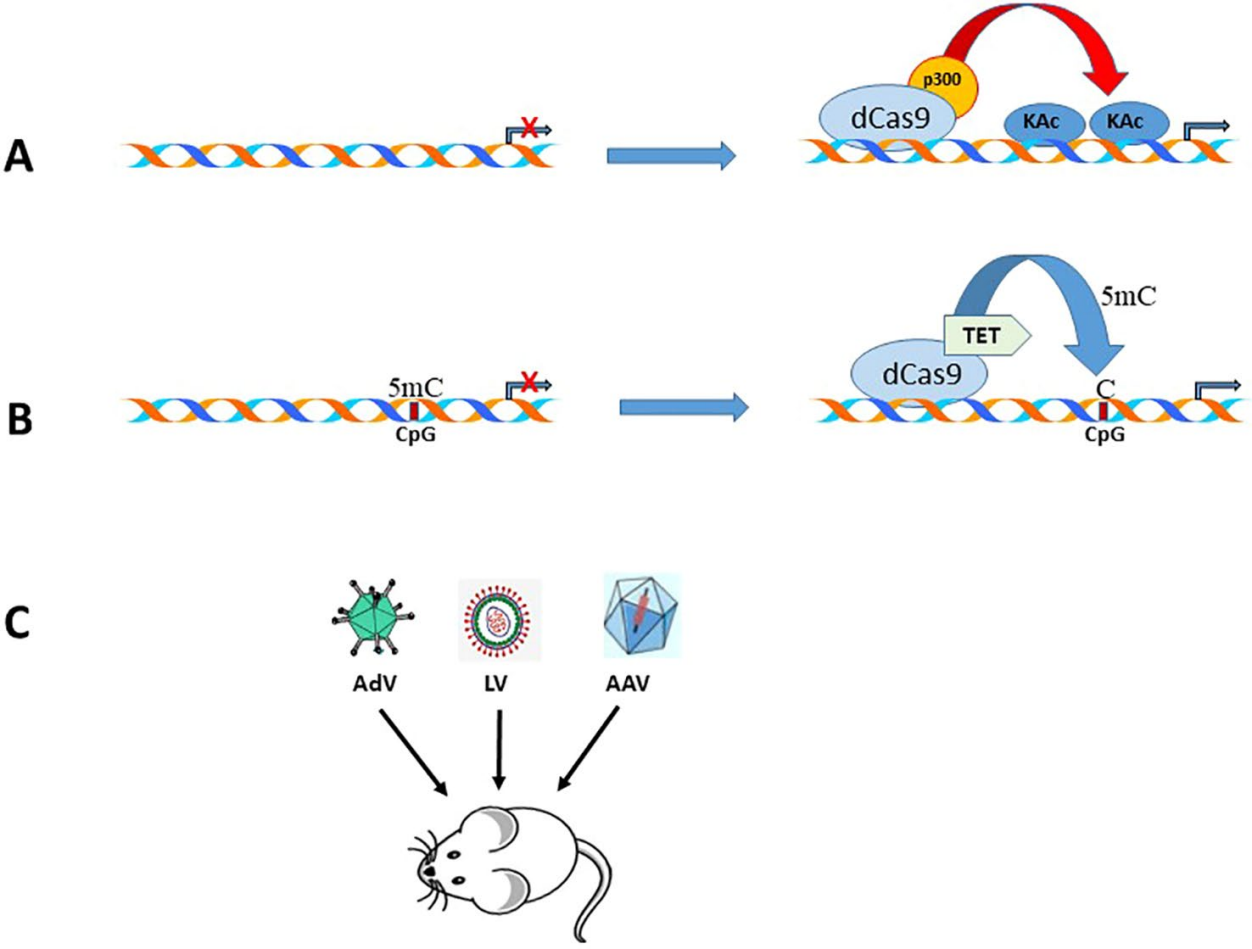
Schematic representation of the CRISPR/Cas9 tools that can be used to activate EAAT2 expression. **(A)**. Epigenome editing by a gRNA, CRISPR-Cas9-based acetyltransferase, dCas9-p300. **(B)**. Epigenome demethylation by a gRNA, CRISPR-Cas9-based demethylase, dCas9-Tet-CD. **(C)**. Strategies for delivery of dCas9/gRNA using different viral vectors in rodent models of neurodegenerative diseases.

With the discovery of several genome editing technologies such as zinc-finger nucleases (ZFNs), transcription activator-like effector nucleases (TALEN), and clustered regularly interspaced short palindromic repeat (CRISPR)/Cas9 system (Datta et al., 2016; Khalili et al., 2017), it is possible not only to edit genes but also activate genes (Hilton et al., 2015) that are epigenetically repressed. Since epigenetic modifying enzymes can be fused to the inactivated dCas9 (D10A mutation in RuvC and H840A in HNH nuclease domain), it is possible to target specific gene promoters using guide RNAs (Tadić et al., 2019) and thereby prevent off-target effects of either overexpression or knockdown of epigenetic modifying enzymes. In the context of EAAT2 gene activation, two CRISPR tools can be used. dCas9 fused to (a) histone acetyltransferase p300 (dCas9-p300) activation domain (Hilton et al., 2015), and (b) DNA demethylase catalytic domain from the TET family (Xu et al., 2016). In the former scenario, the recruitment of dCas9-p300 by guide RNAs can result in histone acetylation mediated EAAT2 gene transcription (**Figure 3A**), and in the latter situation, DNA hypermethylation of the EAAT2 gene CpG islands can potentially be reversed by targeted demethylation of cytosine residues using the Tet catalytic domain (Tet-CD) and guide RNA (**Figure 3B**). This strategy can be accomplished *in vivo* since several viral vectors, including adeno-associated virus, lentivirus, and adenovirus (**Figure 3C**), have been employed for delivery of Cas9 and gRNAs (Gori et al., 2015; Mout et al., 2017).

## Conclusions and Future Directions

A large body of evidence demonstrates the involvement of epigenetic mechanisms, including DNA methylation and histone modification at the pre-transcriptional level and miRNAs at the posttranscriptional level in the dysregulation of EAAT2 expression in numerous neurodegenerative diseases. The mechanisms may also act in concert while regulating EAAT2 expression. The involvement of other epigenetic features, including posttranslational histone modifications, including acetylation, methylation, and phosphorylation, in the regulation of EAAT2/GLT1 promoter activation in astrocytes remains to be investigated in future studies. With the development of new epigenetic drugs with increased sensitivity, specificity, and decreased toxicity it might be possible to upregulate EAAT2 expression in neurological disorders depending on the epigenetic modification that is involved in repression of EAAT2 expression. However, it is likely that in addition to gene-specific modulation, genome wide reactivation or inactivation of genes at random can have potentially deleterious effects. The proposed CRISPR/Cas9 mediated EAAT2 gene regulation can, therefore, be employed in animal models to mitigate glutamate-mediated excitotoxicity.

## Author Contributions

MAA contributed to writing the initial draft of the manuscript and illustrations. PD contributed to writing the review and editing.

## Funding

PD was supported by the National Institutes of Health through grants from National Institute of Drug Abuse, 5R01DA033213, and in part by 5P01DA037830-05.

## Conflict of Interest

The authors declare that the research was conducted in the absence of any commercial or financial relationships that could be construed as a potential conflict of interest.
